# Female bone physiology resilience in a past Polynesian Outlier community

**DOI:** 10.1038/s41598-022-23171-3

**Published:** 2022-11-07

**Authors:** Justyna J. Miszkiewicz, Hallie R. Buckley, Michal Feldman, Lawrence Kiko, Selina Carlhoff, Kathrin Naegele, Emilie Bertolini, Nathalia R. Dias Guimarães, Meg M. Walker, Adam Powell, Cosimo Posth, Rebecca L. Kinaston

**Affiliations:** 1 School of Archaeology and Anthropology, Australian National University, Canberra, Australia; 2 School of Social Science, University of Queensland, St Lucia, Australia; 3 Department of Anatomy, Otago School of Biomedical Sciences, University of Otago, Dunedin, New Zealand; 4 Archaeo- and Palaeogenetics Group, Institute for Archaeological Sciences, University of Tübingen, Tübingen, Germany; 5 Senckenberg Centre for Human Evolution and Palaeoenvironment, University of Tübingen, Tübingen, Germany; 6 Department of Archaeogenetics, Max Planck Institute for Evolutionary Anthropology, Leipzig, Germany; 7 The Solomon Islands National Museum, Honiara, Solomon Islands; 8 Department of Archaeogenetics, Max Planck Institute for the Science of Human History, Jena, Germany; 9 Department of Human Behavior, Ecology and Culture, Max Planck Institute for Evolutionary Anthropology, Leipzig, Germany; 10 Centre for Social and Cultural Research, Griffith University, Southport, QLD Australia; 11 BioArch South, Waitati, New Zealand

**Keywords:** Biological anthropology, Anthropology, Bone

## Abstract

Remodelling is a fundamental biological process involved in the maintenance of bone physiology and function. We know that a range of health and lifestyle factors can impact this process in living and past societies, but there is a notable gap in bone remodelling data for populations from the Pacific Islands. We conducted the first examination of femoral cortical histology in 69 individuals from ca. 440–150 BP Taumako in Solomon Islands, a remote ‘Polynesian Outlier’ island in Melanesia. We tested whether bone remodelling indicators differed between age groups, and biological sex validated using ancient DNA. Bone vascular canal and osteon size, vascular porosity, and localised osteon densities, corrected by femoral robusticity indices were examined. Females had statistically significantly higher vascular porosities when compared to males, but osteon densities and ratios of canal-osteon (~ 8%) did not differ between the sexes. Our results indicate that, compared to males, localised femoral bone tissue of the Taumako females did not drastically decline with age, contrary to what is often observed in modern populations. However, our results match findings in other archaeological samples—a testament to past female bone physiology resilience, also now observed in the Pacific region.

## Introduction

**Figure 1:**
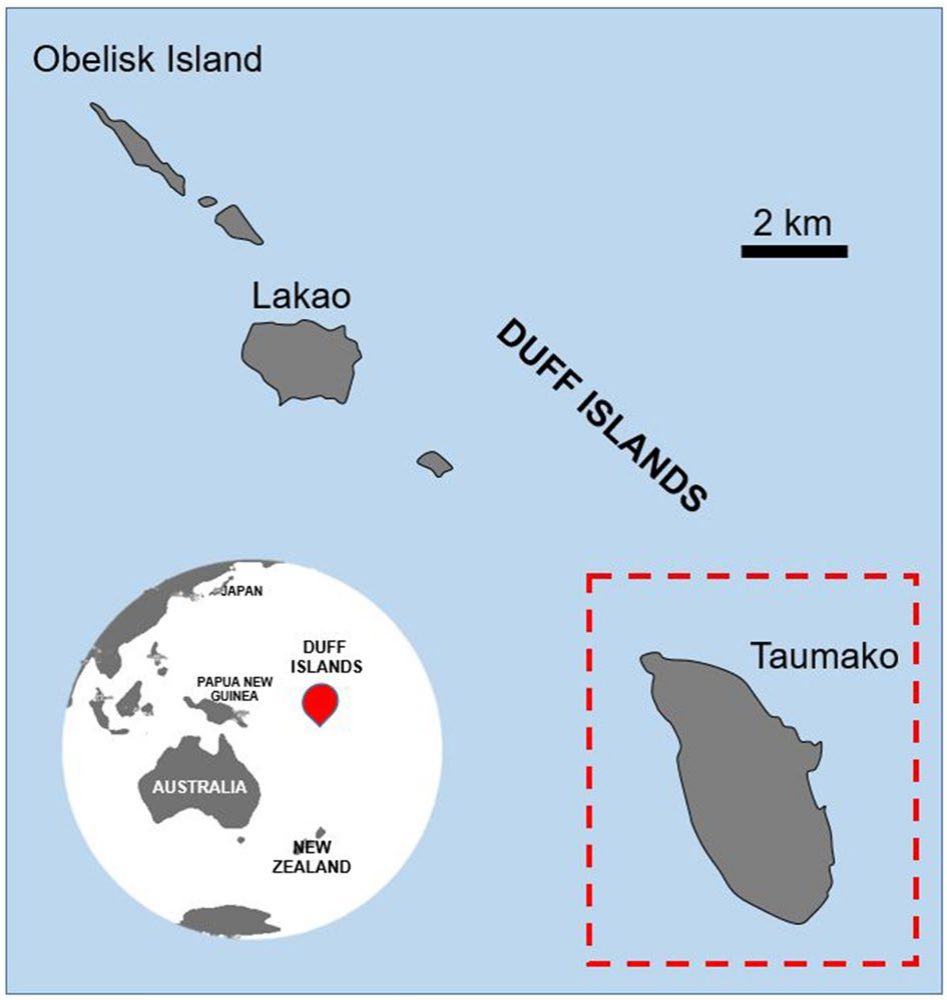
Location of Taumako (red dashed outline), part of the Duff Islands (red marker) complex in Melanesia. Map was drawn by first author (JJM) using Microsoft Office 365 PowerPoint (version 2207) https://www.microsoft.com/en-au/microsoft-365.

Peak bone mass attainment in modern humans occurs around the third life decade and is marked by a striking sex-specific difference whereby biological females (hereafter ‘females’) typically accrue less bone than biological males (hereafter ‘males’)^1–3^. Bone density becomes further compromised around the fifth to sixth life decade when females experience menopause and a significant reduction in the osteoclast inhibiting estrogen^4–6^. The physiological maintenance of bone throughout the life-course is executed by remodelling, a process sensitive to a range of internal and external stimuli^7^. Bioarchaeological research on human skeletal remains with well-preserved bone microstructure has provided data on bone remodelling under a range of cultural and environmental conditions^8–12^. However, there is a notable gap in data for past populations from across the Pacific Islands, except for two recent studies that used small samples sizes quantifying bone vascular porosity in eight individuals from Tonga^13^, and comparing bone histology between the femur, rib, and humerus in one individual from the Marshall Islands^14^. Here, we report the first adult human femur quantitative bone histology data for an archaeological ‘Polynesian Outlier’ skeletal assemblage from a ca. 440–150 BP site on Taumako, Southeast Solomon Islands^15–17^ (Fig. 1).

There are several reasons why bone remodelling in the past inhabitants of Taumako is worth investigating. The Solomon Islands are part of Oceania, which is a region with complex migration histories^18^. Taumako Island, despite being part of the ‘Melanesian’ Solomon Islands, is known as a ‘Polynesian Outlier’ (i.e. part of Polynesia) due to a purported blow-back migration of populations from Polynesia during the mid-second millennium AD, and where Polynesian is the main language today^19^. Human mobility between different regions, including islands from Melanesia, Micronesia, and Polynesia, facilitated an exchange of cultural practices but also encouraged spread of diseases^19,20^. A notoriously high incidence of metabolic and infectious conditions is widespread across the Pacific Islands, particularly in Near Oceania, evidence for which has come from modern epidemiological research and studies of disease in archaeological human remains^21,22^. Although Taumako lies directly east of the boundary with Near Oceania, the archaeological remains from Taumako display a high prevalence of skeletal lesions indicative of endemic yaws and iron-deficiency anaemia (potentially exacerbated by high malaria pathogen loads)^23–25^. This is in addition to large variation in stature, age- and sex-specific dietary practices relating to social status^17,20,26^, and tendency for males to die younger than females at Taumako when compared with neighbouring Tonga in western Polynesia^23,24^. All of these findings suggest experiences of population-wide physiological stress at Taumako. As we do not yet have bone remodelling data for this archaeological sample, we do not understand how, and if, bone growth varied across this population, or whether it was influenced by these experiences of physiological stress. The aim of our study is to report baseline bone remodelling data for Taumako, which will add new insights into the current limited knowledge about past human bone physiology across Pacific Island habitats. Our data will expand understanding of bone growth dynamics within spatially and temporally distributed archaeological populations, and might be of interest to the International Osteoporosis Foundation, which is currently mapping the occurrence of fractures across the Asia–Pacific region^27^.

### Bone remodelling through human life-course

Based on bone mineral density (BMD) and fracture incidence data, it is established that significant bone loss occurs with age^28,29^. Bone building capacities in early adulthood play a key role in determining the rate at which bone metabolic activity becomes out of balance later in life^2^. While early life skeletal mass accrual is largely genetically determined, other factors such as physical activity, diet, and lifestyle habits, can also impact bone metabolism^12,30^. Generally, there are three key areas that characterise bone mass change in modern humans—peak bone mass accrual in the third life decade, drastic bone loss after menopause in females, and significant bone loss in both sexes in old age^2^. The first three life decades are spent creating a ‘bone bank’ that is used for the remainder of the life-course^31^. The female preponderance of bone loss is due to life-course variability in estrogen levels, which inhibit prolonged bone resorption^4^. The effect of menopause on bone health can be mediated through lifestyle factors and calcium supplements available to women today. Modern clinical techniques can diagnose osteoporotic bone from BMD T-scores^32^ and bone remodelling histological markers to check whether osteoclast-mediated bone resorption outweighs bone deposition by osteoblasts.

While BMD has been previously examined in some archaeological samples (see reviews in^8–12,33^), histological characteristics of cortical remodelling assessed from thin sections by histomorphometric and histomorphological methods have also been successfully evaluated^34,35^. Cortical bone not only experiences metabolic turnover events that ensure suitable calcium reservoirs, it also responds to biomechanical stimuli that drive bone cell activity^7^. As teams of osteoblasts and osteoclasts execute bone remodelling as part of Bone Multicellular Units (BMUs) that travel through the cortex, they leave behind remodelling products of circular structure—secondary osteons (hereafter ‘osteons’)—that can be studied histologically, and thus offer an insight into bone remodelling activity in an individual^36^. The area of osteons and Haversian canals within these can aid in determining whether a typical BMU formed over relatively longer or shorter periods of time, whereby larger osteons simply fill more space in bone (though this depends on the ratio of lamellar bone to Haversian canal in individual osteons)^37–40^. Osteons are also replaced by subsequent generations of osteons, creating a total population of remodelled bone per given region^40^, whereas the densities of vascular pores (Haversian canals, Volkmann’s canals, primary/simple vessels) reflect the complex interconnected network cortical bone uses to circulate blood and interstitial fluid containing oxygen and nutrients important for bone homeostasis^41,42^.

### Bone remodelling in archaeological humans

In cases of archaeological human bone that is well preserved microstructurally, studies have been able to reconstruct bone remodelling capacities and link them to aspects such as gender division of labour and sex-specific bone remodelling^43^, changes in subsistence strategies through time^44^, or medieval lifestyles associated with socio-economic disparities^45^. For example, Mulhern and Van Gerven^43^ found higher osteon densities in femoral cross-sections of males than females from Medieval Sudanese Nubia, but no sex differences in Haversian canal dimensions, suggesting sex-specific activities with physically strenuous tasks of males contributing to the observed remodelling patterns. Miszkiewicz et al.^13^ found severely porous Haversian bone in adult females compared to denser bone samples of males from 2650 BP Tonga, indicating experiences of abnormal bone loss likely related to both age and activity. However, bioarchaeological studies where bone remodelling has been investigated through histological means have also cautioned that we do not yet fully understand the spectrum of bone histology parameters manifested in archaeological samples^46^, and that relying on very specific interpretations (e.g. behaviour) made from histological data is clouded by multiple other confounding variables^47^ such as health, nutrition, ancestry and individual or population-based variations in metabolic activity. Therefore, interpretations of archaeological human bone histology data are usually context specific. However, with an increasing number of sites/collections reported, we may be able to start building a better understanding of possible changes in bone remodelling through time and space in recent humans. For example, one prior analysis comparing tibial and femoral bone histology between Pleistocene specimens (including Broken Hill, Shanidar 2, 3, 4, 5, 6, Tabun 1, and Skhul 3, 6, 7) and a pre‐Columbian Pecos human sample, reported similar levels of bone remodelling characterising the two^48^, but smaller size of osteonal structures in the Pleistocene sample^49^.

As bone histology research using archaeological samples gathers increasing amounts of data, it is apparent that a significant gap remains for populations from across the Asia–Pacific region. While access to large samples of human remains is limited in the remote areas of the Pacific, excavation on Taumako Island in the southeast Solomon Islands produced one of the largest well-preserved skeletal samples in the region^15^. Study of this skeletal assemblage presents an excellent opportunity for bone remodelling research.

Modern Pacific Island nations, particularly in Polynesia, are impacted by widespread metabolic syndrome related conditions, including type 2 diabetes and obesity^21^. Archaeological evidence demonstrates the occurrence of gout and diffuse idiopathic skeletal hyperostosis, as well as infectious and nutritional conditions affecting health from the time of first settlement ca 3000 BP in Remote Oceania (the islands east of the main Solomon Islands chain)^21,50–52^. Island environments are associated with food shortages, climate and environmental instability, affecting health in the past and today^21,50,53^. Long-term exposure to pathogens, and population admixture prior to, and crossing-over with, the European contact in the sixteenth to seventeenth centuries could be reflected in community-specific bone remodelling capacities as an adaption to endemic disease and society specific structures that determine diet and society roles. For example, one prior study of 61 Taumako individuals recorded cortical bone indices of the metacarpal and femur, in addition to femur length, to find that no distinct stress or functional adaptation signal could be detected specifically as a result of island conditions^54^. However, this study did not collect microscopic bone data—a gap which our study will fill.

**Figure 2:**
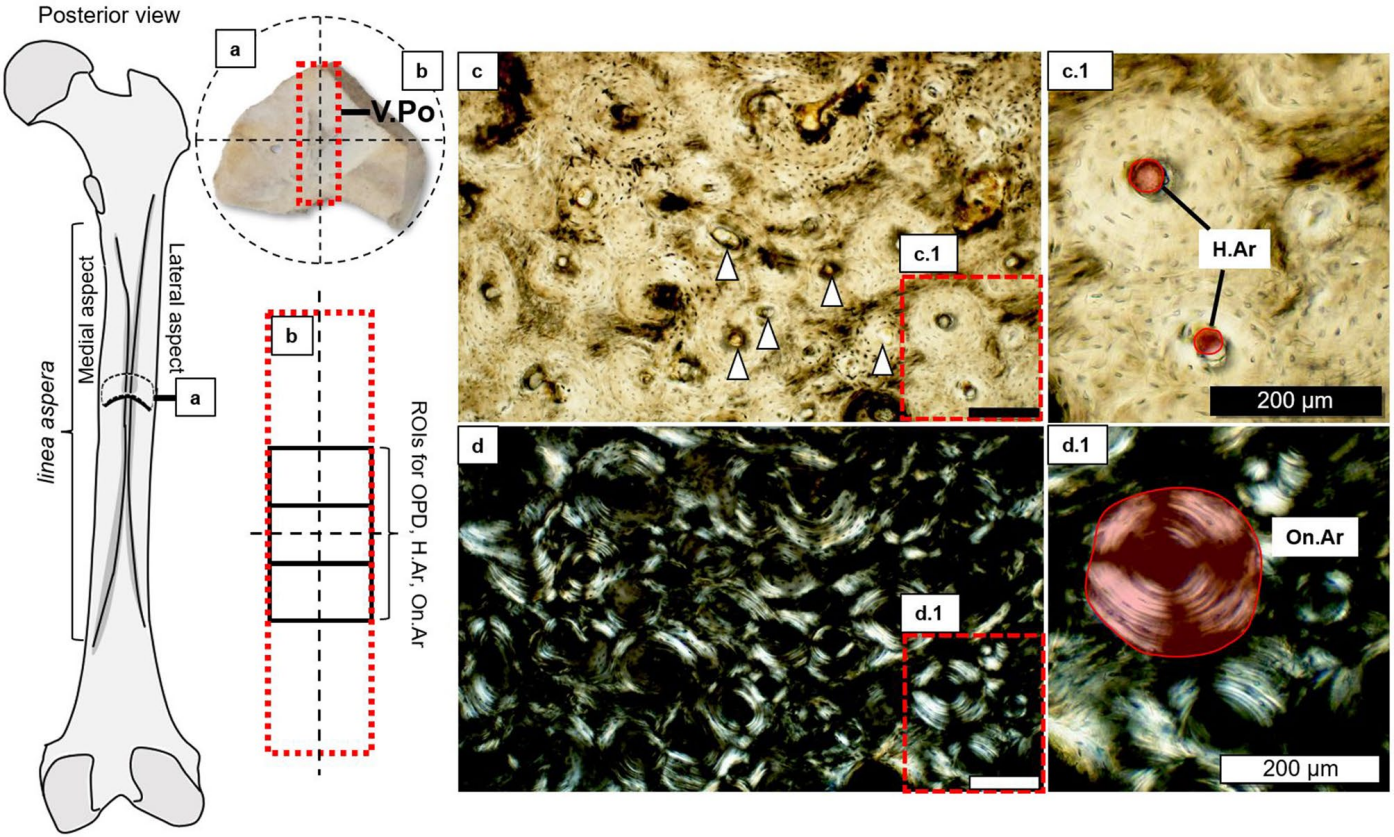
Summary of histomorphometric techniques used in this study. From left sketch of right posterior human femur: (**a**) posterior cortical bone quadrant showing a strip (red dashed lines) of bone surface examined histologically from which vascular porosity (V.Po) was collected; (**b**) three intra-cortical regions of interest (black rectangles) contained within the larger strip examined for osteon population density (OPD), Haversian canal area (H.Ar), and secondary osteon area (On.Ar); (**c**) bone histology under transmitted light showing Haversian canals counted for V.Po (white triangle markers) and measured for area (**c.1**); (**d**) bone histology under linearly polarised light showing secondary osteon area (**d.1**). Scale bars in (**c**,**d**) are 200 μm.

Given the significance of archaeological human samples in improving modern bone biology research, the Pacific Island gap in our knowledge relating to archaeological bone remodelling, and the island environmental context of the Solomon Islands, this study tested whether (1) femur bone histology from archaeological Taumako males and females showed differences in remodelling and tissue organisation indicators, and (2) to what extent these bone microstructure features changed with age. Our total sample size was 69 (33 males and 36 females). We selected the posterior midshaft femur because of its biomechanical versatility reflecting sex-specific lifestyles and sexual dimorphism, which we first evaluated in this sample through basic gross measures of femoral size (midshaft circumference, cortical width, maximum length^54^) and robusticity indices^55^ computed from these values. Next, we created thin sections from which we measured standard static histological variables (vascular canal and osteon area to compute canal-osteon ratios^56^, and localised osteon density^40^) as proxies for bone remodelling activity, and vascular porosity as a proxy for bone blood supply and reflecting bone tissue organisation^41^. Histology was examined in intra-cortical, and combined (including periosteal and endosteal areas) cortical regions of interests (ROIs) of the thin sections (Fig. 2). The femoral size data were then used to adjust histology data to account for microscopic-macroscopic scaling issues, and within-sample variation stemming from inherent bone size differences between males and females. We then compared the data between male and age groups.

We hypothesised indicators of higher bone resorption over formation should be evident in females when compared with males, and in older individuals when compared with those of younger age. Our age and sex estimates are based upon standard gross anatomy methods, which assess age-progressive and sexually dimorphic skeletal landmarks of the skull, teeth, and the pelvis^57,58^. Our sex estimates were validated by determining XY or XX karyotypes via ancient DNA (aDNA), yielding 88% of successful sex matching through these two methodological approaches. This study can only treat sex as a biological trait and cannot consider gender identity which is unknown for these Taumako individuals. We present analyses based on skeletal sexual dimorphism and genetic information within the limits of our sample and available context, but we recognise that many biological traits associated with sex are not binary and exist on a spectrum^59^.

## Results

Sexual dimorphism manifested in size variation across the Taumako femora. The Taumako males had (*p* < 0.001, Tables 1, 2, 3) larger femoral midshafts and thicker posterior cortical walls (average circumference = 95.79 mm, average cortical width = 10.77 mm) compared to females (average circumference = 89.81 mm, average cortical width = 8.77 mm). Females also had slightly shorter femora than males, though this difference was not tested statistically due to a small sub-sample size (n = 23) of the individuals with intact femora. Robusticity indices (unitless values) calculated based upon midshaft circumference and cortical width were greater in males (average circumference robusticity = 22.85; average cortical width robusticity = 2.58) when compared to females (average circumference robusticity = 20.66, average cortical width robusticity = 2.08), but could not be validated using statistical tests either because they were based on the limited femoral length data. We could not apply age related inferential statistical tests to the gross morphometric femoral data, except for circumference and cortical width, which did not change statistically across any of the age classes in the whole sample (*p* > 0.05, Table 1). Within females and males, there was no age effect on midshaft circumference or cortical width either. Given that some of the gross femoral measurements varied with sex (likely because males have larger femora than females in our sample) adjustments of bone histology data by femoral size were necessary^60,61^.

**Table 1:** Descriptive summary of gross femoral data sub-divided by estimated sex and age-at-death. *SD* standard deviation, *MAX.* maximum, *MIN*. minimum, *Ct.W_RI* robusticity index (RI) calculated using cortical width (Ct.W) data, *Circ_RI* robusticity index (RI) calculated using midshaft circumference (Circ). The RI variables are unitless.

Gross femoral measures					
Sub-divided by sex and age-at-death	N	Min	Max	Mean	SD
**Female**					
Femur max length (cm)	6	40.60	44.50	42.42	15.41
Circ (mm)	36	69.00	106.00	89.81	7.86
Ct.W (mm)	36	3.95	13.23	8.77	1.96
Circ_RI	6	17.93	22.33	20.66	1.91
Ct_W_RI	6	1.47	2.57	2.08	0.46
**Male**					
Femur max length (cm)	17	30.40	47.00	42.92	4.70
Circ (mm)	33	85.00	106.00	95.79	5.53
Ct.W (mm)	33	6.56	15.27	10.77	1.65
Circ_RI	17	19.43	34.21	22.85	3.84
Ct_W_RI	17	1.75	4.16	2.58	0.56
**Young adult**					
Femur max length (cm)	16	304.00	477.00	426.81	48.13
Circ (mm)	34	69.00	106.00	91.29	8.05
Ct.W (mm)	34	3.95	15.27	9.61	2.21
Circ_RI	16	17.93	34.21	22.40	4.18
Ct_W_RI	16	1.47	4.16	2.45	0.67
**Middle-aged adult**					
Femur max length (cm)	2	406.00	421.00	413.50	10.61
Circ (mm)	13	81.00	106.00	93.15	7.54
Ct.W (mm)	13	6.64	13.23	9.42	2.07
Circ_RI	2	21.43	22.33	21.88	0.64
Ct_W_RI	2	2.43	2.57	2.50	0.10
**Old adult**					
Femur max length (cm)	5	420.00	458.00	437.00	15.31
Circ (mm)	22	83.00	104.00	94.50	6.15
Ct.W (mm)	22	6.73	13.03	10.07	1.86
Circ_RI	5	19.43	23.80	22.05	1.78
Ct_W_RI	5	2.21	2.78	2.44	0.25

**Table 2:** Descriptive summary of histology data sub-divided by estimated sex and age-at-death groups. *SD* standard deviation, *MAX.* maximum, *MIN.* minimum, *V.Po* density of canals/pores per mm^2^, *H.Ar/On.Ar* ratio of Haversian canal to osteon area in µm^2^, *OPD* osteon population density per mm^2^, *Ct.W_RI* robusticity index (RI) calculated using cortical width (Ct.W) data, *Circ_RI* robusticity index (RI) calculated using midshaft circumference (Circ). All variables are unitless.

Femur histology measures					
Sub-divided by sex and age-at-death	N	Min	Max	Mean	SD
**Female**					
V.Po/Ct.W	36	1.29	4.42	2.34	0.77
V.Po/Circ	36	12.86	39.05	22.02	5.80
H.Ar/On.Ar	9	6.50	10.40	8.31	1.36
OPD/Ct.W_RI	4	6.34	6.84	6.62	0.24
OPD/Circ_RI	4	5.53	7.86	6.76	0.53
**Male**					
V.Po/Ct.W	32	0.83	3.16	1.64	0.49
V.Po/Circ	32	10.46	33.41	18.34	4.49
H.Ar/On.Ar	12	4.57	9.46	7.61	1.52
OPD/Ct.W_RI	8	3.40	6.69	5.31	1.20
OPD/Circ_RI	9	4.78	7.66	6.34	1.08
**Young adult**					
V.Po/Ct.W	33	1.07	4.42	2.02	0.74
V.Po/Circ	33	12.77	29.11	20.37	4.27
H.Ar/On.Ar	13	4.57	9.85	7.83	1.40
OPD/Ct.W_RI	8	3.40	6.69	5.75	1.16
OPD/Circ_RI	9	4.90	7.66	6.44	0.96
**Middle-aged adult**					
V.Po/Ct.W	13	1.29	3.40	2.22	0.69
V.Po/Circ	13	13.98	28.19	21.53	4.75
H.Ar/On.Ar	4	6.50	10.40	8.17	1.83
OPD/Ct.W_RI	1	6.84	6.84	6.84	n/a
OPD/Circ_RI	1	7.86	7.86	7.86	n/a
**Old adult**					
V.Po/Ct.W	22	0.83	3.33	1.88	0.76
V.Po/Circ	22	10.46	39.05	19.43	7.37
H.Ar/On.Ar	4	5.42	9.36	7.92	1.73
OPD/Ct.W_RI	3	4.04	6.81	5.36	1.39
OPD/Circ_RI	3	4.78	7.39	6.10	1.30

**Table 3:** All results of inferential analyses comparing femoral size, and statistically significant results of bone histological markers compared between the sex and age groups. *V.Po* density of canals/pores per mm^2^, *H.Ar/On.Ar* ratio of Haversian canal to osteon area in µm^2^, *OPD* osteon population density per mm^2^, *t* independent samples *t* test, *U* Mann Whitney *U* test, *H* Kruskal–Wallis test, **p < 0.01, ***p < 0.001.

Comparisons	Test statistic	n	*p*
**Males vs. females**			
Circ (mm)	*t* = 3.626	F = 36, M = 33	< 0.001***
Ct.W (mm)	*t* = 4.577	F = 36, M = 33	< 0.001***
**Bone histology markers compared between males and females**			
V.Po/Ct.W (unitless)	*U* = 249	F = 36, M = 32	< 0.0001***
V.Po/Circ (unitless)	*t* = 2.905	F = 36, M = 32	0.005**
H.Ar/On.Ar (unitless)	*U* = 40	F = 9, M = 12	0.345
**Bone histology markers compared between age groups**			
V.Po/Ct.W (unitless)	*H* = 2.4	Y = 33, MA = 13, O = 22	0.301
V.Po/Circ (unitless)	*H* = 3.278	Y = 33, MA = 13, O = 22	0.194

### Femoral vascular porosity and bone remodelling indicators at Taumako

**Figure 3:**
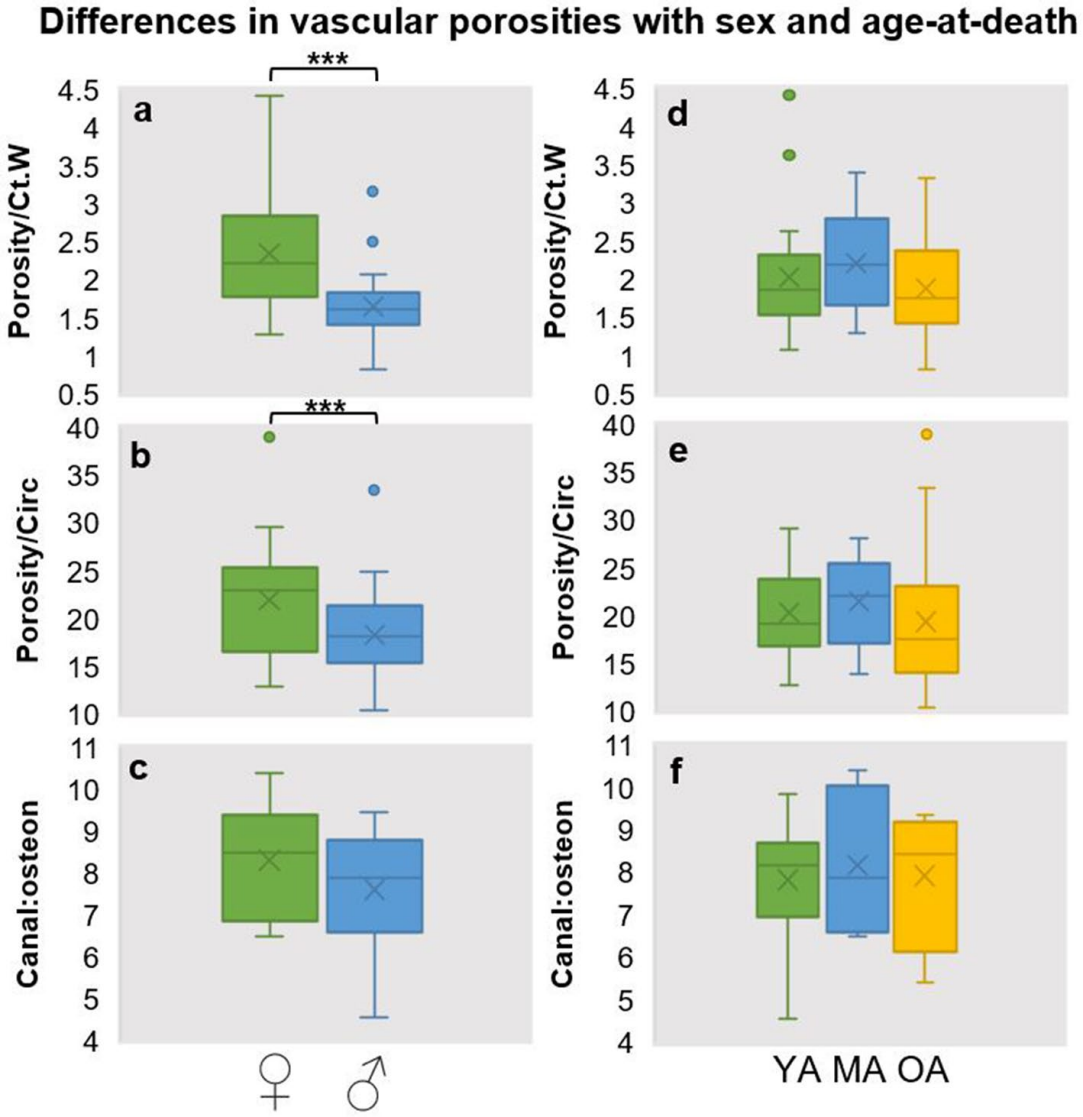
Simple boxplots illustrating differences in vascular porosities adjusted by different measures of femoral bone size (*Circ* circumference of midshaft, *Ct.W* cortical width), and ratio of Haversian canal area to osteon area, compared between the sexes (boxplots **a**–**c**), and age-at-death categories (**d**–**f**; where *YA* young adults, *MA* middle-aged adults, *OA* older adults). ***p < 0.001 using Mann Whitey *U* test (see Table 3).

Looking at descriptive statistics only, vascular porosity, canal-osteon ratios, and osteon densities were greater in females when compared to males (Table 2). When applying inferential statistical tests, out of all three variables, vascular porosity in females adjusted (unitless values) by both cortical width (average vascular porosity adjusted by cortical width = 2.34) and midshaft circumference (average vascular porosity adjusted by midshaft circumference = 22.02) were statistically significantly higher (*p* < 0.0001) than in males (average vascular porosity adjusted by cortical width = 1.64, average vascular porosity adjusted by midshaft circumference = 18.34) (Table 3, Fig. 3). However, the vascular canal-osteon ratios did not differ statistically between the sexes (*p* > 0.05, Tables 2, 3), with both sub-groups bordering an approximate 8% (averages of 7.61% in males and 8.31% in females). We did not attempt an inferential statistical comparison of the osteon density data in the whole sample, or on further sub-divisions by age, due to inadequately small sample sizes (Table 2).

Secondly, there was a clear change in the descriptive statistics of bone histology values from young to old individuals whereby all peaked in the middle-age category (Tables 2, 3, Fig. 3). While all the histology data were lower in the young or old age sub-groups when compared to the middle-age sub-group, the old individuals showed the lowest values across the entire sample with the exception of canal-osteon ratios which were slightly higher. However, none of these changes with age, apparent when considering the data means, were statistically significant (*p* > 0.05) (Table 3). As above, we did not attempt an inferential statistical comparison of the osteon density data in the whole sample, or on further sub-divisions by sex due to inadequately small sample sizes (Table 2). Collectively, our results partly support our hypothesised expectations.

## Discussion

Our age and sex analyses of the Taumako bone histology data revealed that the Taumako females had higher vascular porosity of their femoral cortical bone compared to males, while intra-cortical variables of osteon densities and canal-osteon area ratios did not differ statistically significantly between the sexes. This occurred despite males and females having sexually dimorphic femora at Taumako. A possible isometric effect of larger male femur size on bone histology can be excluded as underlying these results as our data were corrected by femoral midshaft size measures and robusticity indices^60,61^. Acknowledging small sample size in some of the age and sex sub-groups, and with the data at hand, we will discuss possible implications of our results for adult femur bone physiology at Taumako.

### Sex and cortical bone histology at Taumako

The femoral samples of Taumako females were more vascularised than those of males. We will not link this to bone remodelling only^42^, because our data for the vascular porosity are made up of Haversian canals with the possibility of including some primary vessels (see “Materials and methods”). This is a result of us accounting for localised diagenesis apparent in the thin sections. Therefore, this measure is that of an accumulation of vascular cavities up until the point of death, rather than just reflecting recent remodelling events, and we cannot be sure which canals had been replaced in the first few life decades in these individuals. Further, the vascular porosity data stem from the posterior femur ‘strip’ region overlapping an entirety of compact bone, so the porosity counts reflect our inclusion of both the periosteal and endosteal bone regions where there might have been region-specific variation in pore counts. Our main interpretation is that the higher densities of vascular pores in females suggest their bones received greater blood and nutrient supply than that of males^42^. Male frailty due to endemic disease, inferred from their younger mortality compared to females at Taumako^16,26^, may have contributed to this bone characteristic, which we discuss further below. Despite the greater density of cortical pores in females, neither the osteon population density nor the geometric properties of secondary osteons differed statistically between the sexes. The ratio relationship between Haversian canals and osteon area was almost the same when comparing the sexes (approximately 8%). We expected higher Haversian canal area in females than males indicating prolonged osteoclast-mediated bone resorption. This suggests that the intra-cortical midshaft femoral bone in Taumako males and females experienced similar remodelling events.

Prior bioarchaeological research reported inconsistencies in osteon morphometry when comparing the sexes similar to those we present for Taumako. For example, data for males in the Medieval Sudanese sample, mentioned in our Introduction, showed higher osteon densities than in females, but females had larger osteons than males^43^. However, similar to us, Mulhern and Van Gerven^43^ reported a lack of statistically significant differences in the geometric properties of Haversian canals between the sexes. Similarly, fourteenth to nineteenth centuries Pecos females (New Mexico) had relatively large secondary osteons, but with smaller Haversian canals when compared to males^62^. Burr et al.^62^ observed a lack of distinct bone loss in the Pecos females, citing a physically active lifestyle as a possible factor driving the maintenance of good bone density. In the 700 BC to nineteenth century Canadian Baffin Island male and female skeletons, no significant differences were noted when considering the density of Haversian canals and the area of secondary osteons^63^. As noted by Pfeiffer^46^, there is a clear variability in how bone histology is expressed in archaeological populations, as illustrated by the above examples, complicating inter-population comparisons. A Taumako-specific approach is needed to contextualise our results.

Outside of a genetic basis to the morphology of adult compact bone, the Taumako femoral bone histology could reflect a combination of the following population-specific factors: the socio-economic make-up and diet of the community, and the effect of physiological stress and disease on skeletal development. Having studied grave goods (including shell money, bobbles, and Tridacna shell ‘tavi’ neck ornaments) from across the Taumako burials, archaeologists have previously determined that this community was stratified into status groups on the basis of wealth and inherited rank^15^. Leach and Davidson^15^ quantified the value of grave goods finding that the Tridacna breast pendants were the most prestigious. Kinaston et al.^17^ used this information to test for status-related access to food in 99 of Taumako individuals. They^17^ analysed carbon, nitrogen, and sulfur stable isotopes in bone collagen to confirm that wealthy Taumako individuals ate high status foods consisting of high trophic level animals such as pigs, fish, and turtles. This was the case for both high status males and females. However, all wealthy individuals, and all males, overall, had elevated levels of nitrogen when compared to low status females. In our study, at least 15 (~ 22%) individuals were of very high status (Leach and Davidson^15^ used a ‘wealth index’), including nine males and six females, with seven males and five females being buried with the prestigious Tridacna breast pendants. The combination of mixed-sex individuals who regularly fed on high protein foods, and others who fed on lower ranking foods or experienced nutritional stress (see below), could have resulted in the balanced osteon density and canal-osteon areas across the sexes. This mirrors prior research comparing high and low social status human bone histology in medieval England where upper-class foods were associated with higher osteon densities in the femur^45,64^.

In addition, the lack of intra-cortical remodelling differences between the sexes, but lower vascular porosity in males, could be explained through Taumako male frailty. Kinaston and Buckley^16^ used carbon and nitrogen stable isotopes in bone collagen and tooth dentine to infer that nutritional stress led to early deaths of some adolescent and young males at Taumako. This was also found by Stantis et al.^26^ who examined nitrogen stable isotopes in tooth dentine in this sample. Combined with the long history of malaria and yaws exposure at Taumako, experiences of inconsistent dietary intake in lower ranking adolescents might have led to poor bone maintenance later in life^65^ (males are considered to be more susceptible to physiological stress than females because sex-steroids regulate immune response^66,67^). Also, in some indigenous Solomon Island populations today, growth and nutritional status of females is reported to be much better than that of young males^68^. Even though no sex-specific differences in gross anatomical markers of stress, such as linear enamel hypoplasia or lesions indicative of yaws, have been previously noted in the Taumako assemblage^24,69^, our bone histology data offer a microscopic perspective which is a proxy for repetitive, longer-term bone physiological cycles. Thus, we infer that Taumako females might have been equipped with dense intra-cortical femoral bone to buffer excessive bone loss. We know from experimental research that loss of calcium is compensated for by increasing remodelling in lactating females, which ultimately restores compromised bone tissue during reproduction^70,71^.

### The effect of age on bone histology at Taumako

Two key areas of concern to life-long human bone building capacities are the third and fifth-sixth decades reflecting peak bone mass accrual and female menopause, respectively^2^. Bone mass in modern humans through the life-course is easier to map than in past populations as we cannot observe life-long change to bone mass accrual in the archaeological record. However, our sample size is large enough to begin unravelling Taumako bone remodelling differences across the three anthropological age categories. The entire sample followed an expected trend in secondary bone change with age, whereby both osteons and vascular porosity increased through the lifespan in mature individuals^72,73^. Further, all the bone histology data appeared to peak at the middle-age category, which mirrors the expectation based on modern bone health through the life-course paradigms. We acknowledge such comparison cannot be exact given the broad age anthropological categories, but the end of the young, and the start of the middle-age age category, overlaps with the peak age for bone mass accrual in living humans^2^. When considering intra-cortical osteon densities and canal-osteon ratios, it becomes apparent that Taumako females maintain similar localised amounts of bone as males across different age categories. This aligns with the same age-related observation in 1250–1450 AD Sudanese Kulubnarti, Nubia, where no statistically significant changes in the geometric parameters of osteons were observed^43^. Our data also match some of the findings reported by Burr et al.^62^ for the Pecos females who appeared to maintain good bone well into adulthood, and any age-related reduction in bone quality was that of marrow cavity expansion in both males and females. Generally, a smaller secondary osteon size has been previously noted to occur with age in both males and females today^73^, which applies to our results, and is similar to previous reports for the Pecos males^62^.

A preliminary and cautious comment about bone histology in our old males and females can also be made. Although osteons were only measured in four well-preserved histology samples, representing four different individuals, all had densely remodelled Haversian bone. There was no indication of early stages of osteoporosis, including cortical bone trabecularisation or the presence of ‘giant’ coalescing pores^13^. Further, osteon density data in the one old female matched osteon density data for the female middle-age category, and were higher than the combined osteon density data in all three old male samples. We cannot exclude the effect of osteon population density asymptote on the data in the old category, whereby the evidence of pre-existing secondary osteons may have been erased by subsequent generations of remodeled bone^74^. However, we can build a hypothesis, worth testing in future bioarchaeological research, whether bone histology from old archaeological females exhibits severe porosity (in the sense of trabecularisation, not just vascular counts) intra-cortically^75^. This would help in validating to what extent, and at what age, past human female long bone cortex develops osteoporosis in the fifth life decades or later. While most anthropological methods of age estimation do not provide specific chronological ages, or decades, some studies have recognised it might be possible to separate individuals aged 50 + years old into further age classes^76,77^. Histological sampling in such instances could contribute to this vein of research wherein older individuals could be gradually examined decade by decade (similar to modern efforts, e.g.^75^). Although, a detailed contextual information and burial/population background^78^, along with permissions for destructive sampling, would be needed.

Our study highlights the significance of combining gross anatomical and microscopic approaches to understanding bone biology in archaeological contexts. Robb et al.^54^ reported some effect of age on metacarpal cortical bone indices, and femoral length, in the Taumako sample without accessing microstructural indicators of cortical bone remodelling. Our robusticity indices were calculated for femora instead of just reporting length, and followed a robusticity methodological recommendation based on a published thorough technical evaluation of different robusticity measures^55^. It will be important for future bioarchaeological studies to combine macro- and microscopic technical approaches as limb bone size and shape determined through ontogenetic modelling completes after the first two life decades^79^. While modelling declines for the remainder of the lifespan, and re-activates in extreme biomechanical situations, bone remodelling information can be only accessed microscopically.

## Remarks on temporal and spatial bone histology data

We acknowledge that bone histology interpretations in archaeological settings need to be conducted at a population level, but given our study presents the first osteon remodelling data for the Pacific region, we can establish that the Taumako data fall into a global range of secondary osteon parameters for archaeological humans^38,43,45,62,63^. Some examples include: the Taumako male and female combined average osteon area (28,433 μm^2^) data are similar to 27,303 μm^2^ reported for medieval Canterbury, England^38,45^; the male and female combined area of Haversian canals in Taumako is 2221 μm^2^ which compares closely to 2100 μm^2^ in medieval (1250–1450 AD) Sudanese Kulubnarti, Nubia^43^, 2336 μm^2^ in fourteenth to nineteenth centuries Pecos, New Mexico^62^, and 2334 μm^2^ in medieval Canterbury, England^38,45^. Similarities can also be noted in raw osteon density data, whereby the Taumako data of 13.64/mm^2^ are close to 11.78/mm^2^ in Sudanese Nubia^43^. We acknowledge the above studies used slightly different region of interest (ROI) selection techniques, but all considered femoral midshaft cortical bone. Future bone histology research on archaeological specimens spanning other geographical regions will expand this range.

## Limitations

We cannot exclude a series of confounding factors that have impacted our results and interpretations. The estimates of age and sex for a portion of the sample at Taumako rely on anthropological standards, as such they are probability scores. However, the aDNA validation of the bulk of the sex estimates in this study overcame some of the uncertainty of gross methods. Age assessments were validated as much as possible by ensuring that each individual’s histology profile generally matched its age status established from the gross anatomical methods (e.g. thin sections were inspected for possible presence of primary bone in samples from older adults). Unfortunately, we cannot overcome the inconsistencies in sample size in each age and sex sub-group either, and do not have access to better preserved bone histology. It must be said that some of the statistically insignificant results could simply be a result of sampling given the specimens available to us, which is an issue for all bioarchaeological studies. Finally, we only use two-dimensional methods of thin sectioning, but a wider volumetric dataset providing three-dimensional perspectives on vascularity connectedness, in combination with mineral density information, would provide a more in-depth picture of bone building and remodelling capacities in the Taumako sample.

## Conclusions

The Pacific islands are yet to be thoroughly studied for past human bone histological variation. Our study forms the first, largest sample size based, report of archaeological human intra-cortical secondary osteon, and cortical vascular porosity, data in this part of the world. We found that archaeological females at Taumako show highly vascularised femoral midshaft bone, but also have localised areas of intra-cortical bone that remodels similarly to that of males. This finding mirrors bone remodelling data from other archaeological sites from across North America, Europe, and Africa, but does not conform entirely to our modern understanding of bone loss through the life-course. These new data fall in the range of bone histology archaeological variability reported globally, extending currently available bone histology data by this site from the Pacific Islands. The lower vascular porosity in males might reflect their higher frailty in the cultural and environmental context of Taumako, and the balanced remodelling indicators between the sexes could be a result of socially stratified dietary practices. Ongoing efforts examining bone histology in Asia–Pacific will further our understanding of ancient human bone remodelling capacities in this region, contributing to modern efforts investigating the conditions under which humans experience significant bone loss.

## Materials and methods

Taumako is one of the remote Duff Islands, which lie northeast of Santa Cruz Islands in the far southeast Solomon Islands^15^ (Fig. 1). While the island is located within the Melanesian geographical boundary, Taumako is known as a ‘Polynesian Outlier’ representing a probable ‘blow-back’ migration of populations from Polynesia around the mid-second millennium AD^15,80,81^. The modern inhabitants of Taumako speak a Polynesian language, but as a result of admixture with established populations, share similar cultural traditions to nearby Melanesian islands in the Duff and Santa Cruz groups^82^.

The Namu burial mound, an archaeological site dated to ca. 440–150 BP^20^, yielded a significant number of human remains and associated grave goods that have been since examined to reconstruct the lives of the past inhabitants of Taumako. This has included social status stratification^15,17^ reflected in dietary and child feeding practices reconstructed from bone and tooth stable isotope data^16,17,26,83^; abnormalities of the alveolar bone suggesting possible experiences of periodontitis^84^; evidence for interpersonal violence and warfare inferred from skeletal patterns of trauma^85^; a high prevalence of yaws (*Treponema pertenue*)^24^; and, more recently, gender specific migration patterns from neighbouring islands^20^. The site is known for archaeological evidence of using shell money and ornamentation practices that include a *tavi*—a neck ornament thought to represent high status (see^15^). Another study also analysed Taumako femur length and metacarpal and femur cortical indices and noted a lack of distinct bone functional adaptation in remote Pacific Island environments^54^.

With permissions from, and in collaboration with, the Solomon Islands National Museum, n = 69 Taumako adults were sampled in the present study. There were 19 left and 50 right femora. Both sides were pooled due to no statistically significant bilateral differences in the recorded data (Supplementary Information Tables 1, 2). Following standard methods recommended by Buikstra and Ubelaker^57^, and Brickley and McKinley^58^, each individual was thoroughly examined for skeletal markers of sex, and those that change with age, to arrive at morphologically informed biological sex and age-at-death (we use ‘age’ in the main article) estimates. The age categories follow these standard recommendations whereby individuals are assigned into ‘young’ (20–35 years old), ‘middle-aged’ (36–50 years old) and old (50 + years old) age-at-death classes. As is good practice in bioarchaeology, for each individual, as many techniques of examination were applied as possible to increase the accuracy of the estimates. These methods involve a gross anatomical examination of the following: dental wear of permanent teeth with higher degrees of wear progressing with age; obliteration degree of cranial sutures which progressively close with age; texture and general morphology changes of the pelvic auricular surface and the pubic symphysis, which disintegrate with age. Each skeletal technique gives an independent age range, which are then compiled into common ranges that can be placed into the major age-at-death classes. We have no way of corroborating the anthropological estimates, which are necessarily broad, with actual chronological age of these individuals, which is unknown.

The biological sex estimation was based upon examining the skull and pelvic anatomical landmarks which are known to be sexually dimorphic. A non-exhaustive list of these features includes: the robusticity level of the mastoid process; the nuchal crest of the occipital bone; the shape of the eye orbits and the thickness of the orbital roof; the prominence of the glabella; and the mental eminence of the mandible; the angle of the pelvic sciatic notch; the presence or absence of the pelvic ventral arc, subpubic concavity, and a medial ridge in the pubis region.

Skeletal morphology is more robust in the male skeletal remains, though we acknowledge this is a generalisation. Thus, unlike with our age-at-death estimates, with permission from the Solomon Islands Museum, we were able to validate the sex estimates through aDNA obtained from genome-wide data that had been produced for a subset of samples investigated histologically. Individuals were sampled for DNA by drilling the petrous part of the temporal bone^86^ (see dataset^87^). DNA was extracted from the sampled powder and prepared for next-generation sequencing by producing a double-stranded DNA library following established protocols^88–90^. Deaminated cytosines were enzymatically partially removed and retained only in the terminal positions as described in^91^. All libraries were directly shotgun sequenced on an Illumina HiSeq 4000 platform (1 × 75 + 8 + 8 cycles). The sequenced reads were mapped to the human genome reference hg19 using EAGER^92^. The retained damage was excluded from the analysis by masking the two terminal positions of each read^93^. The genetic sex was inferred using two independent methods:

1. The number of reads covering each position was counted across a total of around 1.24 million genome-wide SNP positions^94–96^ and subsequently averaged for each sex chromosome and all autosomal ones. The Y- and the X-chromosome average coverages were normalized by the average autosomal coverage and compared to determine the sex assignment^97^.
2. An approach specifically designed for low-covered shotgun genomes in which the ratio between the average coverage across the entire X-chromosome and the coverage averaged across the autosomes was calculated as in^98^ (see Supplementary Information for extended aDNA methods). This was possible for n = 48 individuals. There was an 88% success rate (42/48) in corroborating the macroscopic and aDNA sex results, with only six individuals misclassified by the gross methodologies (see Supplementary Information Table 3). Therefore, the presented sex classification can be treated as fairly reliable. We acknowledge we do not attempt to classify these as ‘gender’, but treat them as a biological entity in relation to bone metabolic processes. As a result, this study comprised 34 young adults, 13 middle-aged adults, 22 old adults, and 36 females and 33 males. Further sub-division by age-at-death within each sex group can be seen in the dataset^87^.

Prior to histological analyses we recorded a series of femur morphometric measurements to characterise the size of each femoral midshaft and calculate femoral robusticity indices where possible^55,60^. Three variables were included: midshaft circumference (Circ) in mm, posterior cortical width (Ct.W) in mm, and femur maximum length in cm. These were measured using standard osteological laboratory equipment composed of an osteometric board, digital calipers, and a soft measuring tape. Two robusticity indices were computed using the Stock and Shaw^55^ recommendation and following prior methods combining femoral bone histology and robusticity measures^45^: femoral robusticity index based on Circ where the circumference values are divided by femoral length, and a femoral robusticity index based on Ct.W where cortical width values are divided by femoral length and multiplied by 100. The latter included multiplication by 100 to increase decimals in the resulting robusticity index values for the ease of our statistical analysis. Only 23 femora were of a suitable preservation for measuring the maximum length, and so only these were used in the robusticity index calculations.

Next, posterior cortical bone samples from the midshaft of each femur were extracted using a Dremel tool with a rotary blade, resulting in approximately 1 cm thick cortical quadrants (see^45^). The posterior femur is of interest to our study because it overlaps the *linea aspera*, a rich leg muscle site insertion anatomical landmark. Bone remodelling detected there should capture stimulation resulting from lifestyle^99,100^, which will strengthen our analyses of age and sex. Our minimal invasive approach ensured the femora remained as intact as possible, limiting the amount of archaeological bone being taken for the histological analysis^101^. Standard histological methods relevant to archaeological human remains were then followed to produce ~ 100 μm thin sections^34^. Each sample was embedded in Buehler epoxy resin, cut using a Kemet MICRACUT precision cutter equipped with a diamond blade, glued to a microscope slide, further reduced, ground, and polished to obtain a clear view of bone histology. The thin sections were examined using an Olympus BX53 microscope with a DP74 camera using transmitted and linearly polarised light at a magnification of 10 × (100 × total magnification).

Once histology slides were prepared, it became apparent that not all microstructures could be measured in all sections. Well preserved ROIs where cement lines of secondary osteons were easily identifiable were the case for only 21 individuals, but 68 individuals had consistently and suitably preserved Haversian canals. A diagenetically obscured band that ran along the outer posterior and endosteal layers of bone samples was also observed in the thin sections. However, the intra-cortical regions of bone were of an almost pristine preservation, which allowed us to focus on intra-cortical remodelling activity away from the immediately sub-periosteal and sub-endosteal regions of cortical bone. As such, we designed the ROI selection procedure so that data can be collected from the mid-portion of each sample by scanning a full cortical strip down the midline and then capturing three ROIs within its centre (Fig. 2). The examination of cortical strips as ROIs, and intra-cortical bone regions generally, were successful in prior archaeological studies^44,102^.

We used the Olympus cellSens software (“Standard” version 2018, https://www.olympus-lifescience.com/en/software/cellsens/), which allows to automatically stitch images in live scanning mode. This function was used to record each ROI ‘strip’. A thin section was placed on the microscope stage so that the mid-point of the periosteal border was in the field of view. The stage was then slowly moved forward (away from the observer) until the endosteal end of the border was reached. The area of the ROI strips ranged from 6.76 to 25.84 mm^2^ in our sample given variation in cortical wall thickness (mean = 14 mm^2^, standard deviation = 3.67 mm^2^). From within the strip, the first ROI was located at the mid-point (by dividing the length of the entire strip by two), and then one ROI was taken either side of this midpoint, ensuring no overlap in histology shown in the field of view (Fig. 2). Using FIJI/ImageJ tools that included the “Multi-Point Count” and “Polygon” selections, three histomorphometric variables indicative of cortical bone remodelling events were measured (we use bone histomorphometry nomenclature recommended by Dempster et al.^103^ Fig. 2):

- **Vascular porosity** (V.Po) per mm^2^ (e.g.^13,62,104,105^): total number of intact Haversian and primary canals across a full strip ROI of bone measured from the posterior to the endosteal borders of the section, and divided by the strip area in mm^2^. Volkmann’s canals were excluded because they were rarely visible in the sample. Because we worked with archaeological specimens and 2D histology sections, true vascular porosity, including other minute capillaries is not possible to obtain. In instances where cement lines of osteons were not visible, we cannot be entirely confident that a counted canal derives from a secondary osteon structure. As such, we use V.Po to represent all major vascular canals seen in the ROI strip.
- **Osteon population density** (OPD) per mm^2^ (e.g.^43,45^): sum of intact osteon and fragmentary osteon numbers counted from three intra-cortical ROIs of 2.05 mm^2^ area each (totalling 6.15 mm^2^). Each sum was divided by the ROI area in mm^2^.
- **Haversian canal:osteon area ratio** (H.Ar/On.Ar), measured in μm^2^ separately, and then converted to unitless (dimensionless variable (DV)) ratio values (e.g.^45,56,60^): H.Ar is the average area (total area/total number of measured canals) of intact Haversian canals measured from three intra-cortical ROIs of 2.05 mm^2^ area each (totalling 6.15 mm^2^); On.Ar is the secondary osteon area in μm^2^ created from average area (total area/total number of measured secondary osteons) of intact secondary osteons with complete cement lines measured from three intra-cortical ROIs of 2.05 mm^2^ area each (totalling 6.15 mm^2^). Secondary osteons cut off by an image border were excluded. The average values of H.Ar are then divided by the average values of On.Ar and multiplied by 100 to indicate percentage of canal to osteon area.

Recommended standards for reporting of bone histomorphometric data stipulate a minimum of 25 osteons examined per thin section^40^. Our study meets those standards by examining a minimum of 47 and maximum of 126 secondary osteons across the samples for the purposes of osteon density calculations, and minimum 25 and maximum 50 for the purpose of ratio calculations from area measurements of osteon units. The V.Po and OPD data are used in our study as products of bone remodelling events that indicate the amount of bone produced and remodeled per mm^2^ intra-cortically^45^. The area of Haversian canals and secondary osteons can be used as indicators of the stage of a BMU travelling through the cortex^36^. Larger areas of osteons and canals can be associated with longer periods of BMU activity, and smaller areas would indicate a shorter-term BMU activity, particularly if it is strain-suppressed^38,106^.

**Figure 4:**
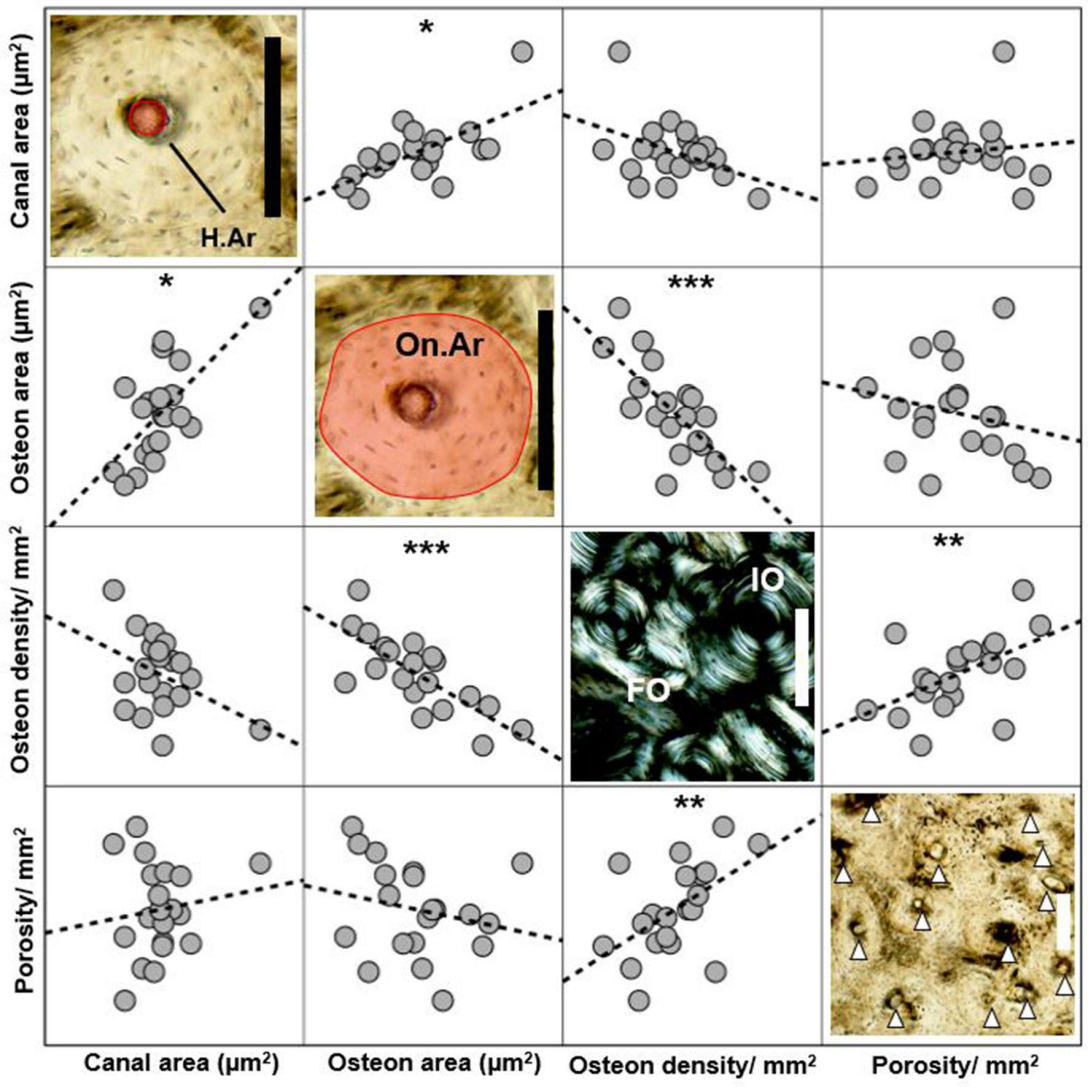
Montage combining simple correlations between the key histomorphometric variables examined in this study. We do not show y and x axis values as this figure is intended as a simple illustrative overview of how well the variables agree with each other. *p < 0.05, **p < 0.01, ***p < 0.001 using Spearman’s *Rho* tests (see Supplementary Information Table 4).

Prior to addressing the main questions of our study, we ran non-parametric Spearman’s *Rho* tests (due to sample size smaller than 30 in at least one sub-group that was being included in the correlations) correlating all the histology variables to check whether porosities, densities, and area measures increased or decreased in values when considered alongside each other. This step was necessary as we have different histology data from two different types of ROIs (the ‘strip’ and three localised ROIs within), so we wanted to check that each variable can be treated independently in our interpretation. Statistically significant relationships, and those of *Rho* > 0.35^107^, were taken to indicate that the variables reflected expected relationships such as allometry between osteon and canal area, and the density variables^60^. The histology correlations returned three statistically significant and strong relationships (Fig. 4; Supplementary Information Table 4) for the area of osteons and their Haversian canals (positive correlation), osteon area and population density (negative correlation), and vascular porosity and osteon population density (positive correlation). The area of osteons increased as the area of canals increased, higher osteon densities were associated with smaller osteons (which is expected for a strained posterior femur), and higher vascular porosities corresponded to higher osteon densities. This information means that despite collecting different histology data from different ROIs, all related to one another statistically allowing us to interpret them all in the comparisons with age and sex.

Next, the V.Po and OPD variables were adjusted by the previously measured midshaft variables and calculated RIs to account for a possible isometric relationship between femur size and the underlying histological structures^61^. It is possible that larger femora could simply show higher values of canals and osteons as a result of inherent size variation across the sample. This was also important as previous research indicated that sexually dimorphic bones may still build bone tissue of similar quality^108^.

The H.Ar/On.Ar ratio variable did not require adjustments as it is in itself already a quantitative relation between two histology measures of size. The V.Po variable was adjusted by raw Circ and Ct.W (creating V.Po/Circ, and V.Po/Ct.W), whereas OPD was adjusted by robusticity index (Circ) and robusticity index (Ct.W) where the femoral maximum length was available for robusticity index calculations.

A brief descriptive analysis summarising data using mean, minimum, maximum, and standard deviation (SD) values was conducted in first instance. The quantitative variables in n > 30 (Circ, Ct.W, V.Po, V.Po/Ct.W, V.Po/Circ) were tested for normality using the Kolmogorov–Smirnov test. Parametric tests were then selected for normally distributed variables (Circ, Ct.W, V.Po, V.Po/Circ), and non-parametric tests were applied to V.Po/Ct.W where data were not normally distributed. For data in sub-groups of n < 30, non-parametric inferential tests were selected without normality tests given the sample size. As a result, Mann–Whitney *U* tests or *t* tests were applied when comparing bone macro- and microstructure between the sexes. When comparing the three age-at-death groups, we used a non-parametric Kruskal–Wallis test with a post-hoc pairwise comparison. For the gross femoral analyses we report significant results only, whereas for the histology analyses we show all results because they are interpreted to answer our research questions. We did not run statistical analyses on the OPD data, and age-at-death and sex sub-divisions due to inadequate sample sizes in the sub-groups.

### Ethics approval

Approval to conduct this research was obtained from the Solomon Islands National Museum. The analysis and release of data were in consultation with, and co-authorship by, community representative (Lawrence Kiko), with whom also a report summarising the findings was filed prior to discussing publication. The thin sections will be repatriated to the Solomon Islands National Museum upon the completion of this project. All research followed ethical guidelines of the American Association of Biological Anthropologists and the Australasian Society for Human Biology.

## Supplementary Information

Supplementary Information.

## Data Availability

Data are available open access from Figshare (Miszkiewicz et al. 2022^87^) 10.6084/m9.figshare.16815295.
