# Supplementary material for: Female bone physiology resilience in a past Polynesian Outlier community
Source: Sci Rep. 2022 Nov 7;12:18857. doi: 10.1038/s41598-022-23171-3 (PMC9640697; doi:10.1038/s41598-022-23171-3)
Supplement: Supplementary file 1 — Supplementary Information. [file 41598_2022_23171_MOESM1_ESM.pdf]

SUPPLEMENTAL INFORMATION FOR

Female bone physiology resilience in a past Polynesian outlier community

Justyna J. Miszkiewicz[1,2*], Hallie R. Buckley[3], Michal Feldman[4,5,6], Lawrence Kiko[7], Selina Carlhoff[6], Kathrin Naegele[6], Emilie Bertolini[8], Nathalia R. Dias Guimarães[1], Meg M. Walker[1], Adam Powell[9], Cosimo Posth[4,5,6], Rebecca L. Kinaston[3,10,11]

[1]School of Archaeology and Anthropology, Australian National University, Canberra, Australia
[2]School of Social Science, University of Queensland, St Lucia, Australia
[3]Department of Anatomy, Otago School of Biomedical Sciences, University of Otago, Dunedin, New Zealand
[4]Archaeo- and Palaeogenetics Group, Institute for Archaeological Sciences, University of Tübingen, Tübingen, Germany
[5]Senckenberg Centre for Human Evolution and Palaeoenvironment, University of Tübingen, Tübingen, Germany
[6]Department of Archaeogentics, Max Planck Institute for Evolutionary Anthropology, Leipzig, Germany
[7]The Solomon Islands National Museum, Honiara, Solomon Islands
[8]Department of Archaeogentics, Max Planck Institute for the Science of Human History, Jena, Germany
[9]Department of Human Behavior, Ecology and Culture, Max Planck Institute for Evolutionary Anthropology, Leipzig, Germany
[10]Centre for Social and Cultural Research, Griffith University, Queensland, Australia
[11]BioArch South, Waitati, New Zealand

***CORRESPONDENCE**: j.miszkiewicz@uq.edu.au (ORCID: 0000-0002-9769-2706)

SI Table 1. Descriptive summary of data differences between the left and right femora. SD: standard deviation, SE mean: standard error mean.

VARIABLES	FEMUR SIDE	N	MEAN	SD	SE MEAN
Femur length (mm)	Right	14	426.86	43.05	11.50
	Left	9	429.44	39.55	13.18
Midshaft circumference (Circ) (mm)	Right	50	92.32	8.26	1.17
	Left	19	93.58	4.63	1.06
Posterior cortical width (Ct.W) (mm)	Right	50	9.48	2.20	0.31
	Left	19	10.37	1.52	0.35
Vascular porosity (V.Po) adjusted by Ct.W (unitless) (V.Po/Ct.W)	Right	50	2.10	0.78	0.11
	Left	18	1.77	0.52	0.12
V.Po adjusted by Circ (unitless) (V.Po/Circ)	Right	50	20.47	5.75	0.81
	Left	18	19.77	4.87	1.15
Haversian canal:secondary osteon area ratio (H.Ar/On.Ar) (unitless)	Right	14	8.08	1.24	0.33
	Left	7	7.57	1.89	0.72
Osteon population density (OPD) adjusted by Ct.W robusticity index (Ct.W_RI) (OPD/Ct.W_RI)	Right	8	5.87	1.23	0.43
	Left	4	5.50	1.14	0.57
OPD adjusted by Circ robusticity index (Circ_RI) (OPD/Circ_RI)	Right	8	6.39	1.04	0.37
	Left	5	6.59	1.16	0.52
Circ_RI	Right	14	22.48	3.91	1.04
	Left	9	21.97	3.08	1.03
Ct.W_RI	Right	14	2.43	0.65	0.17
	Left	9	2.47	0.45	0.15

SI Table 2. Summary of data differences between the left and right femora tested using an independent samples *t*-test. There were no statistically significant differences in all variables, so both femoral sides were pooled for our analyses. DF: degree of freedom, DIFF.: difference, SE: standard error.

VARIABLES	*t*	DF	*p*	MEAN DIFF.	SE DIFF.	95% CONFIDENCE INTERVAL OF THE DIFF.	
						LOWER	UPPER
Femur length mm	-0.145	21	0.886	-2.59	17.84	-39.68	34.51
Circ mm	-0.626	67	0.534	-1.26	2.01	-5.27	2.76
Ct.W mm	-1.606	67	0.113	-0.88	0.55	-1.98	0.21
V.Po/Ct.W	1.640	66	0.106	0.33	0.20	-0.07	0.72
V.Po/Circ	0.463	66	0.645	0.70	1.52	-2.34	3.74
H.Ar/On.Ar	0.743	19	0.466	0.51	0.68	-0.92	1.94
OPD/Ct.W_RI	0.502	10	0.627	0.37	0.74	-1.27	2.01
OPD/Circ_RI	-0.320	11	0.755	-0.20	0.61	-1.56	1.16
Circ_RI	0.329	21	0.745	0.745	0.51	1.54	-2.70
Ct_W_RI	-0.146	21	0.885	0.885	-0.04	0.25	-0.55

EXTENDED DNA METHODS

The DNA processing was conducted in dedicated aDNA facilities at the Max Planck Institute for the Science of Human History, Jena, Germany. For each individual, the dense part within the petrous portion of the temporal bone was drilled for DNA sampling [1]. DNA was extracted from around 50 mg of the sampled powder following published protocols [2]. To prepare the extract for next-generation sequencing a 25-ul aliquot was processed to produce a double-stranded and double-indexed Illumina DNA library following [3, 4]. To prevent that post-mortem deamination damages would be mistaken as authentic sequences in downstream analysis, damage caused by cytosine deamination was partially removed using uracil-DNA glycosylase and endonuclease VIII as described in [5]. Damage was retained in the two terminal positions to be later used for estimating the fraction of deaminated reads [5]. The DNA libraries were subsequently amplified using Herculase II Fusion DNA polymerase according to the manufacturer's protocol. All libraries were directly shotgun single-end sequenced on an Illumina HiSeq 4000 platform (1 × 75 + 8 + 8 cycles). To control for potential laboratory contamination, blank extractions and library preparations were included for each sample batch.

The sequenced reads were binned (demultiplexed) allowing for one mismatch per index. The multiplexed libraries were than processed using the EAGER (v 1.92.54) pipeline [6]. As part of the pipeline, the Illumina adapter sequences were clipped off and the reads were filtered, retaining only reads longer than 30 base pairs using AdapterRemoval (v2.2.0) [8]. The clipped and filtered reads were mapped against the human genome reference hg19 using the BWA aln/samse alignment software (v0.7.12), with a stringency parameter of 0.01, seeding off (-l 16,500), and only retaining reads with Phred-scaled mapping quality scores higher than 30 [8]. Duplicate reads were removed using DeDup v0.12.2 [6]. To authenticate the ancient DNA library, levels of DNA deamination post-mortem damage were measured using mapDamage (v2.0) [9] and compared to the expected values in similar libraries prepared from ancient skeletal elements. Two terminal positions of each fragment were then masked to exclude DNA damage from following analyses [10].

Due to the low number of sequences yielded for each library, the genetic sex was inferred using two independent approaches. Both approaches aim to determine the copy number of each sex chromosome by calculating the number of reads mapping to sex- and autosomal chromosomes. Since genetic females have two copies of the X-chromosome and two copies of each autosomal chromosome, their X-chromosome coverage is expected to be comparable to the autosomal one. However, males have only one copy of the X-chromosome and one of the Y-chromosome and therefore the coverage of each of their sex chromosomes is expected to be half of the autosomal one.

The first method uses the mapping counts across a total of around 1.24 million genome-wide SNPs that were ascertained since they are informative for population history studies [11-13]. However, they can also be useful to estimate genetic sex [14]. For this purpose, the reads mapping to each ascertained position were counted using SAMtools and averaged for each chromosome using an inhouse script [15]. The Y- and the X- chromosome average coverages were each normalized using the average autosomal coverage. Then the normalized Y- and the X- chromosome average coverages were compared and used for the sex assignment.

The second approach was specifically designed for low-covered shotgun genomes and has been shown to confidently estimate genetic sex for libraries with as little as 1,000 mapping reads. In contrast to the first method, here the average coverage is estimated across the entire X- and the entire autosomal- chromosome sequences of the human reference hg19 (and not

on specific positions). The ratio between the X and the autosomal average coverages is calculated and used for the sex assignment as described in [16].

References:

1. Pinhasi, R. *et al.* DNA yields from the inner ear part of the human petrous bone. *PLOS ONE* **10**, e0129102 (2015).
2. Dabney, J. *et al.* Complete mitochondrial genome sequence of a Middle Pleistocene cave bear reconstructed from ultrashort DNA fragments. *Proc. Natl. Acad. Sci. U.S.A.* **110**, 15758–15763 (2013).
3. Meyer, M., Kircher, M. Illumina sequencing library preparation for highly multiplexed target capture and sequencing. *Cold Spring Harb. Protoc.* **2010**, pdb.prot5448 (2010).
4. Kircher, M., Sawyer, S., Meyer, M. Double indexing overcomes inaccuracies in multiplex sequencing on the Illumina platform. *Nucleic Acids Res.* **40**, e3 (2012).
5. Rohland, N., Harney, E., Mallick, S., Nordenfelt, S., Reich, D. Partial uracil–DNA–glycosylase treatment for screening of ancient DNA. *Philos. Trans. R. Soc. Lond. B Biol. Sci.* **370**, 20130624 (2015).
6. Peltzer, A. *et al.* EAGER: Efficient ancient genome reconstruction. *Genome Biol.* **17**, 60 (2016).
7. Schubert, M., Lindgreen, S., Orlando, L. AdapterRemoval v2: Rapid adapter trimming, identification, and read merging. *BMC. Res. Notes* **9**, 88 (2016).
8. Li, H., Durbin, R. Fast and accurate short read alignment with Burrows–Wheeler transform. *Bioinformatics* **25**, 1754–1760 (2009).
9. Jónsson, H., Ginolhac, A., Schubert, M., Johnson, P.L.F., Orlando, L. mapDamage2.0: Fast approximate Bayesian estimates of ancient DNA damage parameters. *Bioinformatics* **29**, 1682–1684 (2013).
10. Jun, G., Wing, M. K., Abecasis, G. R., Kang, H. M. An efficient and scalable analysis framework for variant extraction and refinement from population-scale DNA sequence data. *Genome Res.* **25**, 918-925 (2015).
11. Fu, Q. *et al.* DNA analysis of an early modern human from Tianyuan Cave, China. *Proc. Natl. Acad. Sci. U.S.A.* **110**, 2223–2227 (2013).
12. Haak, W. *et al.* Massive migration from the steppe was a source for Indo-European languages in Europe. *Nature* **522**, 207–211 (2015).
13. Mathieson, I. *et al.* Genome-wide patterns of selection in 230 ancient Eurasians. *Nature* **528**, 499–503 (2015).
14. Fu, Q., *et al.* The genetic history of ice age Europe. *Nature* **534**, 200-205 (2016).
15. Li, H. A statistical framework for SNP calling, mutation discovery, association mapping and population genetical parameter estimation from sequencing data. *Bioinformatics* **27**(21), 2987-2993 (2011).
16. Mittnik, A., Wang, C. C., Svoboda, J., Krause, J. (2016). A molecular approach to the sexing of the triple burial at the Upper Paleolithic Site of Dolní Věstonice. *PloS one*, **11**, e0163019.

SI Table 3 (continues p. 6). Matching of sex results based on gross anatomical methods and those supported by aDNA. There was total n = 69, total n of mismatches = 6, total n of matches = 42, which results in 88% success rate of sex estimation using both methods. aDNA was not available (n/a) for n = 21 individuals.

**ID**	**Estimated sex**	**Genetic sex approach 1 (all positions; X/auto ratio)**	**Genetic sex approach 2 (1240K positions; X/Y ratio)**	**Mismatch**
B178	Female	Male	Male	x
B54	Female	Female	Female	
B83	Female	n/a	n/a	n/a
B91	Female	Female	Female	
B13	Female	n/a	n/a	n/a
B140	Female	Female	Female	
B21	Female	Male	Male	x
B48	Female	Male	Male	x
B63	Female	Female_low_certainty	Female	
B115	Female	Female	Female	
B121	Female	Male	Male	x
B150	Female	Female	Female	
B84	Female	Female	Female	
B141	Female	n/a	n/a	n/a
B139	Female	Female	Female	
B3	Female	Female	Female	
B41	Female	n/a	n/a	n/a
B71	Female	n/a	n/a	n/a
B103	Female	Female	Female	
B163	Female	Female	Female	
B159	Female	Female_low_certainty	Female_low_certainty	
B6	Female	n/a	n/a	n/a
B65	Female	Female	Female	
B79	Female	n/a	n/a	n/a
B23	Female	n/a	n/a	n/a
B15	Female	Male	Male	x
B25	Female	n/a	n/a	n/a
B38	Female	n/a	n/a	n/a
B109	Female	Female_low_certainty	Female	
B59	Female	Female	Female	
B152	Female	Female	Female	
B37	Female	n/a	n/a	n/a
B110	Female	Female	Female	
B160	Female	n/a	n/a	n/a
105-1	Female	n/a	n/a	n/a
105-2	Female	n/a	n/a	n/a
B180	Female	Female	Female	
B30	Female	Female	Female	
B45	Female	n/a	n/a	n/a
B95	Female	Female	Female	
B69	Female	Female	Female	

B44	Female	Male	Male	x
B149	Male	Male	Male	
B68	Male	Male	Male	
B195	Male	n/a	n/a	n/a
B108	Male	Male	Male	
B42	Male	Male	Male	
B73	Male	Male	Male	
B85	Male	n/a	n/a	n/a
B126	Male	Male	Male	
B145	Male	Male	Male	
B169	Male	Male	Male	
B148	Male	n/a	n/a	n/a
B177	Male	Male	Male	
B179	Male	Male	Male	
B194	Male	n/a	n/a	n/a
B1	Male	Male	Male	
B181	Male	Male	Male	
B104	Male	Male	Male	
B176	Male	Male	Male	
B189	Male	Male	Male	
B196	Male	Male	Male	
B24	Male	n/a	n/a	n/a
B133	Male	Male	Male	
B87	Male	Male	Male	
B14	Male	Male	Male	
B173	Male	n/a	n/a	n/a
B182	Male	Male	Male	
B22	Male	Male	Male	

SI Table 4. Statistically significant results of correlations of bone histological markers compared between the sex and age-at-death groups. H.Ar: Haversian canal area, On.Ar: osteon area, OPD: osteon population density per mm^2, V.Po: density of canals/pores per mm^2, *Rho*: Spearman's Rho test. *$p < 0.5$, **$p < 0.01$, ***$p < 0.001$.

**CORRELATIONS**	**Test statistic**	**n**	***p***
**Histology correlations across the sample**			
H.Ar and On.Ar	*Rho* = 0.435	21	0.049*
On.Ar and OPD	*Rho* = -0.670	21	<0.001***
V.Po and OPD	*Rho = 0.531*	20	0.016**
